# Direct Bio-Utilization of Untreated Rapeseed Meal for Effective Iturin A Production by *Bacillus subtilis* in Submerged Fermentation

**DOI:** 10.1371/journal.pone.0111171

**Published:** 2014-10-31

**Authors:** Hu Jin, Xinran Zhang, Kunpeng Li, Yanxing Niu, Mian Guo, Chuanjiong Hu, Xia Wan, Yangmin Gong, Fenghong Huang

**Affiliations:** 1 Oil Crops Research Institute, Chinese Academy of Agriculture Sciences, Wuhan, China; 2 Hubei Key Laboratory of Lipid Chemistry and Nutrition, Wuhan, China; 3 College of Life Science, Hubei University, Wuhan, China; Glasgow University, United Kingdom

## Abstract

The feasibility of using untreated rapeseed meal as a nitrogen source for iturin A production by *Bacillus subtilis* 3–10 in submerged fermentation was first evaluated by comparison with two different commercial nitrogen sources of peptone and ammonium nitrate. A significant promoting effect of rapeseed meal on iturin A production was observed and the maximum iturin A concentration of 0.60 g/L was reached at 70 h, which was 20% and 8.0 fold higher than that produced from peptone and ammonium nitrate media, respectively. It was shown that rapeseed meal had a positive induction effect on protease secretion, contributing to the release of soluble protein from low water solubility solid rapeseed meal for an effective supply of available nitrogen during fermentation. Moreover, compared to raw rapeseed meal, the remaining residue following fermentation could be used as a more suitable supplementary protein source for animal feed because of the great decrease of major anti-nutritional components including sinapine, glucosinolate and its degradation products of isothiocyanate and oxazolidine thione. The results obtained from this study demonstrate the potential of direct utilization of low cost rapeseed meal as a nitrogen source for commercial production of iturin A and other secondary metabolites by *Bacillus subtilis*.

## Introduction

The increasing demand for a steady, healthy food supply requires an efficient control of major plant pests and diseases [1]. Currently, conventional crop management practices are based largely on the application of synthetic pesticides, which have caused serious environmental and health problems. Moreover, the efficacy of synthetic pesticides is gradually decreasing due to the adaptation and resistance of pathogens to these pesticides [2]. One of the greatest ecological challenges in the near future is to develop environmentally friendly alternatives [3]. Recently, biological control agents have received considerable attention as alternatives to chemical pesticides for plant diseases and are considered to be one of the most promising methods for rational and safe crop-management practices [4]. *Bacillus subtilis*, one of the most commonly used and well-studied microbial species, has the potential to produce more than two dozen structurally diverse antimicrobial compounds with broad inhibitory spectrum and high viability [5]. Among these antimicrobial compounds, cyclic lipopeptides of iturin, surfactin and fengycin families have well-recognized potential uses in biotechnology and biopharmaceutical applications because of their excellent surfactant properties.

Iturin A is a cyclic lipopeptide antibiotic with the structure of a cyclic heptapeptide linked to a 14–17 carbons β-amino fatty acid chain [3]. This special structure endows it with strong antifungal action because of its membrane permeable properties [6]. It is a potential weapon for controlling plant diseases caused by phytopathogenic fungi. However, a significant obstacle to large-scale industrial application of cyclic lipopeptide antibiotics such as iturin A is the high production cost coupled with a low production rate. If the production costs of these cyclic lipopeptide antibiotics become competitive with the synthetic chemical pesticides, their industrial use might be expected to grow tremendously in the coming decade [7]. In order to achieve this goal, recent efforts have been focused on the reduction of lipopeptide antibiotics production costs through improving the yield and utilization of low-cost feedstocks, such as soybean curd residue [8], soybean and sweet potato residues [9], cassava flour wastewater [10], [11], synthetic wastewater [12] and waste soybean oil [13]. However, no reports comparing the production performance of lipopeptide by using these feedstocks with that by using common industrial fermentation raw materials have been found.

Rapeseed meal is the by-product of oil extraction from seeds and is mainly composed of proteins, fibres and minerals. In 2011, the production of rapeseed meal was estimated at about 35 million tons [14]. In view of their large quantities, the effective utilization of these residual meals is becoming a crucial issue which should be considered. Traditionally, rapeseed meal is used as organic fertilizer and animal feed due to its high protein content. However, the utilization of rapeseed meal in feed industries is limited by some anti-nutritional or toxic constituents such as phytic acid, sinapine, fiber and glucosinolate [15], [16]. Furthermore, compared to other protein rich waste materials such as soybean meal, rapeseed meal proteins are not easily digestible, which makes them less valuable [17]. However, rapeseed meal might be used as an inexpensive, eco-friendly fermentative nutrient for the growth of microorganisms owing to its high content of protein, carbohydrate and mineral [18]. Originally, microbial treatments were applied to help detoxification and improve rapeseed meal's digestibility and functional properties for animal feeding via solid-state fermentation [16]. More recently, new alternatives to these traditional uses of rapeseed meal have emerged to yield the high value-added compounds of industrial interest through fermentation processes. Unfortunately, as the rapeseed meal nutrients could not be directly assimilated by the majority of industrial bacteria and yeasts, at present, direct bioconversion of rapeseed meal has been mainly limited to the application of filamentous fungi with solid state fermentation. Compared to solid state fermentation, submerged fermentation has been more widely applied in industrial fermentation processes because it has the advantages of high homogeneity of heat and mass transfer, easy control of oxygen and temperature, better monitoring and more convenient handling [19], [20]. Except for a few studies related to pretreated rapeseed meal as substrate for bio-products production by submerged culture fermentation [17], [19], [21], there are no reports about the direct utilization of rapeseed meal as a renewable resource for cyclic lipopeptide antibiotics production through microbial submerged fermentation. In fact, pretreatment of rapeseed meal increases the production cost and even causes the loss or damage of some important rapeseed meal nutrients. Therefore, direct bio-utilization of rapeseed meal is a more promising and practical production mode.

The objective of this study was to investigate the feasibility of using untreated low cost rapeseed meal as nitrogen source to produce valuable cyclic lipopeptide antibiotics iturin A by *Bacillus subtilis* 3–10 in submerged fermentation. Subsequently, the production performance of iturin A and fermentation process properties using rapeseed meal as nitrogen source were compared with other commercial nitrogen sources in bioreactor batch fermentation. Moreover, the possibility of rapeseed meal for the improvement of iturin A yield was analyzed. Finally, the distribution and degradation characteristics of the major rapeseed meal toxic components in both supernatant and residue before and after fermentation were evaluated.

## Materials and Methods

### Microorganism

The iturin A production strain of *Bacillus subtilis* 3–10 (GeneBank accession number JF460845), isolated from a soil sample collected at suburb field of Wuhan, was kindly provided by Professor Shouwen Chen (State Key Laboratory of Agricultural Microbiology, Huazhong Agricultural University, Wuhan, China).

### Medium

The LB medium used for seed culture had the following composition (in g/L, unless otherwise specified): Tryptone 10, Yeast extract 5, NaCl 10, and 20 g/L agar was added to the slant medium. The fermentation medium was composed of (in g/L) Glucose 20, K_2_HPO_4_·3H_2_O 1, MgSO_4_·7H_2_O 0.5, MnSO_4_·H_2_O 0.005, and Rapeseed meal 90. The initial pH of the medium was adjusted to 7.0 and autoclaved at 121°C for 30 min.

### Culture conditions

For seed preparation, *Bacillus subtilis* 3–10 from a fresh slant was inoculated into 30 mL seed medium in 250 mL flasks and cultured in a rotary shaker at 220 rpm for 12 h. Bioreactor experiments were performed in a 7 L bench-scaled bioreactor (BIOSTAT A Plus, Sartorius Stedim Biotech, Germany) with the initial working volume of 3 L. The inoculation size was 5% (v/v). All fermentations were carried out at 28°C and pH was maintained automatically at approximately 7.0 by the addition of 4 M NaOH or 4 M H_2_SO_4_ solutions. Aeration and agitation rates were maintained at 2 vvm and 600 rpm, respectively. During the fermentations, samples were taken at 4–12 h intervals for off-line analysis.

### Analytical methods

#### Determination of viable cell number

Viable cell number during submerged fermentation was determined as follows: 0.5 mL of sample was placed into a sterile 10 mL test tube, and then the sample was mixed thoroughly with 4.5 ml of sterile distilled water and shaken at 150 rpm using a vortex for 5 min at room temperature. The mixture was then serially diluted and spread onto LB-agar plates. After 24 h of incubation at 28°C, the number of colonies was counted and expressed as colony forming units (CFU).

#### Extraction and quantitation of iturin A

Iturin A was extracted according to a reported method [8] with some modifications: 300 µL of strain 3–10 culture was suspended in a microtube containing 1,200 µL of methanol and shaken for 60 min. The mixture was centrifuged at 12,000 rpm for 20 min, and the supernatant was filtered through a 0.22-µm pore-size hydrophobic polytetrafluoroethylene (PTFE) type disposable syringe. The iturin A concentration in the filtrate was quantified with a Waters 2695 HPLC system equipped with a reversephase high-performance liquid chromatography (HPLC) column (ACQUITY UPLC BEA C18 1.7 µm 2.1×100 mm, Waters, USA) at a flow rate of 0.3 mL/min. A mixture of acetonitrile and 10 mM ammonium acetate (35∶65, v/v) was used as the eluent and the elution was monitored at 210 nm. The concentration of iturin A was analyzed and quantified using the standard iturin A assay (Sigma Chemicals, St. Louis, MO). The contents of iturin A at different time points were measured from triplicate samples.

#### Measurements of reducing sugar and protein concentrations

The fermentation samples were centrifuged at 12,000 rpm for 20 min, and the reducing sugar and soluble protein concentration in the supernatant was measured. The reducing sugar concentrations were determined by DNS method using 3, 5-dinitrosalicylic acid reagent [22]. Soluble protein concentrations in supernatant were determined by the Bradford method using bovine serum albumin as a standard [23]. The total nitrogen/protein contents in rapeseed meal raw material and residue samples were measured by a Kjeldahl method using a protein analyzer (2300 Kjeltec Analyzer Unit, ACTAC, Japan). The protein content was calculated from measured total nitrogen using a conversion factor of 6.25.

#### Assays of protease and cellulase activities

Protease activities were assayed by determining the increase in free amino nitrogen (FAN) concentrations that were produced during hydrolysis of 10 g/L casein (Merck) at 40°C in 200 mM, pH 7.5 phosphate buffer. One unit of protease activity (U) was defined as the protease required for the production of 1 µg FAN in one minute [24]. The carboxymethyl cellulase (CMCase) activities were assayed using 0.8% carboxymethyl cellulose as the substrate [25]. Supernatants from three replicate cultures were analyzed for enzyme activity. Next, 1.5 mL of supernatant was mixed with 0.5 mL of 1% carboxymethyl cellulose (CMC) solution in 0.5 M potassium phosphate buffer (pH 5.5). The reaction proceeded for 1 h at 37°C, and was stopped by boiling for 5 min. Boiled samples were centrifuged at 12,000 rpm for 5 min, and the reducing sugar produced in the supernatants was measured colorimetrically by using the dinitrosalicylic acid method [22].

#### Assays of crude fiber, sinapine and glucosinolate, as well as isothiocyanate and oxazolidine thione

Crude fiber was determined according to the method described by Vig et al [16]. Sinapine concentration was determined with a rapid high-performance liquid chromatographic method [26]. After centrifugation, the supernatant and residue were freeze-dried to constant weight for glucosinolate, isothiocyanate and oxazolidine thione measurements. Glucosinolates content was determined according to the palladium chloride method [27]. The quantitative analysis of isothiocyanate and oxazolidine thione was performed as previously described [28].

#### Statistical analysis

Student's t-test was employed to evaluate these data, and samples with P<0.05 were considered significantly different.

## Results and Discussion

### Influence of initial glucose and rapeseed meal concentrations on iturin A production in shake flasks

The dissolution characteristic of rapeseed meal is similar to soybean meal, a widely used slow release nitrogen source in industrial fermentation processes [29]. During submerged fermentation, their nutrients are partly solubilized, gradually released and utilized. The reported optimal soybean meal concentration for iturin A production was 80 g/L in submerged fermentation when it was used as a nitrogen source [20]. Therefore, to test the availability of rapeseed meal nitrogen by *Bacillus subtilis* 3–10, an initial rapeseed meal concentration of 90 g/L was adopted to keep similar nitrogen content with soybean meal in shake flasks experiments.

**Figure 1:**
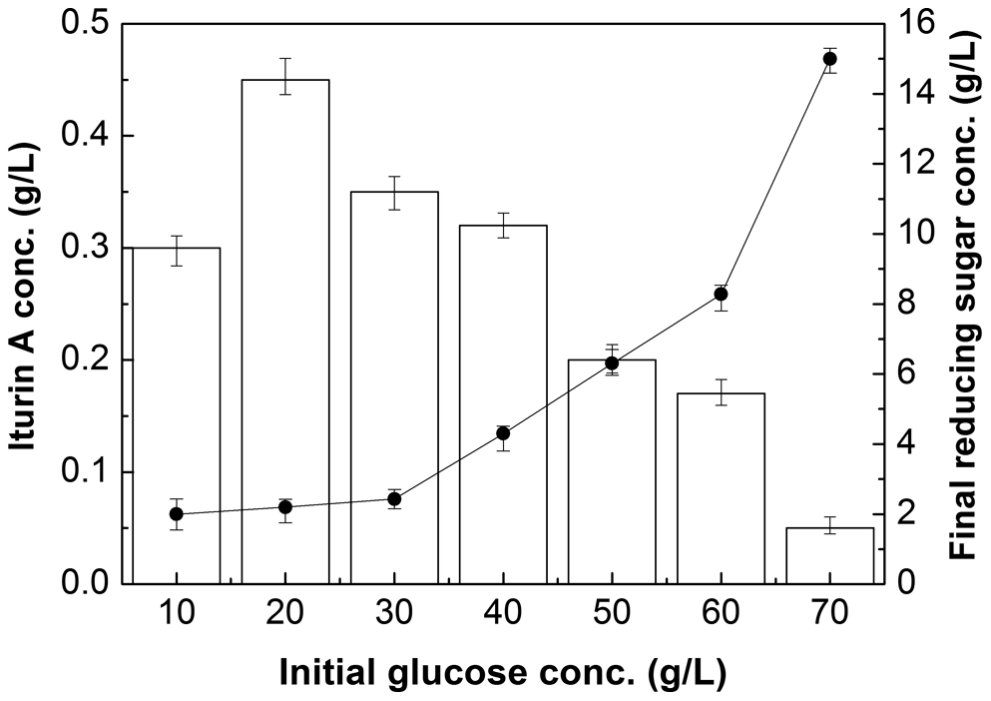
Effects of different initial glucose concentrations on iturin A production and final reducing sugar concentrations at 72 h in shake flasks. ▪: reducing sugar concentration. Error bars represent the standard deviation of the means (N = 3).

The effect of different initial glucose concentrations ranging from 10 to 70 g/L on iturin A production in shake flasks is in Figure 1. It was clearly demonstrated that untreated rapeseed meal can be used as a nitrogen source for iturin A production. The optimal initial glucose concentration for iturin A production was 20 g/L, and the corresponding iturin A concentration reached 0.45 g/L at 72 h. With the increase of initial glucose concentration from 30 to 70 g/L, the production of iturin A decreased gradually, showing that high initial glucose concentration was not suitable for iturin A production during the entire fermentation period. Interestingly, after 72 h culture, the final reducing sugar concentrations were very similar (about 2 g/L) when the initial glucose was added between 10 and 30 g/L (Figure 1), indicating that the remaining reducing sugars in the supernatant may be unavailable and could not be consumed by *Bacillus subtilis* 3–10. Moreover, higher initial glucose concentrations (40–70 g/L) resulted in a higher amount of final reducing sugar up to 15 g/L when the initial glucose concentration was 70 g/L (Figure 1).

**Figure 2:**
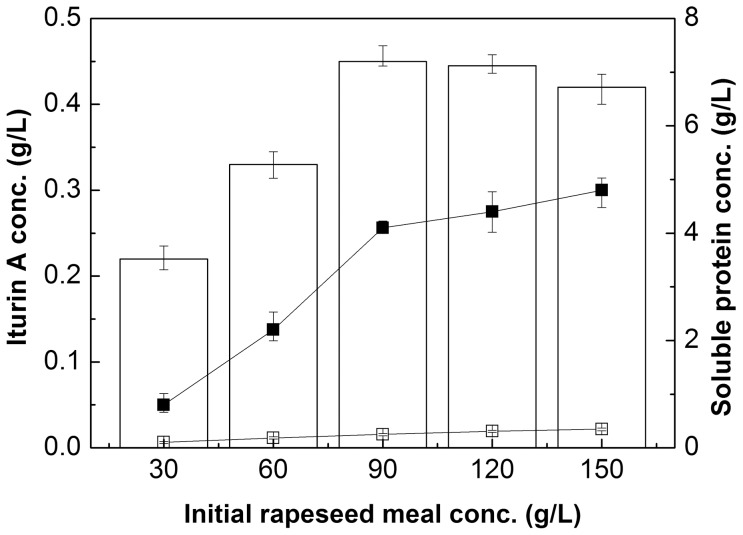
Effects of different initial rapeseed meal concentrations on iturin A production and the initial and final soluble protein concentrations with glucose concentration of 20 g/L in shake flasks. □: initial soluble protein concentration; ▪: final soluble protein concentration. Error bars represent the standard deviation of the means (N = 3).

Subsequently, the effect of different initial rapeseed meal concentrations on iturin A production was investigated under the above optimal glucose concentration of 20 g/L. As shown in Figure 2, the optimal initial rapeseed meal concentration for iturin A production was 90 g/L. When the initial concentration of rapeseed meal increased from 30 to 90 g/L, the iturin A concentration increased from 0.22 to 0.45 g/L accordingly; however, the continued increase of initial rapeseed meal from 90 to 150 g/L resulted in the decline of iturin A concentration. Only 0.5–0.8% initial protein was dissolved in the supernatant (Figure 2). Moreover, with the increase of initial rapeseed meal concentrations, the initial soluble protein increased correspondingly (from 0.10 to 0.35 g/L while the initial rapeseed meal increased from 30 to 150 g/L). Further, after 72 hours of fermentation by *Bacillus subtilis* 3–10, the soluble protein concentration increased significantly compared to the initial concentrations. The soluble protein at 72 h reached 4.8 g/L and increased about 13.7 fold when the initial rapeseed meal concentration was 150 g/L, indicating that a large amount of protein from rapeseed meal were released and dissolved into the medium by the effect of *Bacillus subtilis* fermentation (Figure 2).

### Significant advantage of rapeseed meal on iturin A production in bioreactor batch fermentation

**Figure 3:**
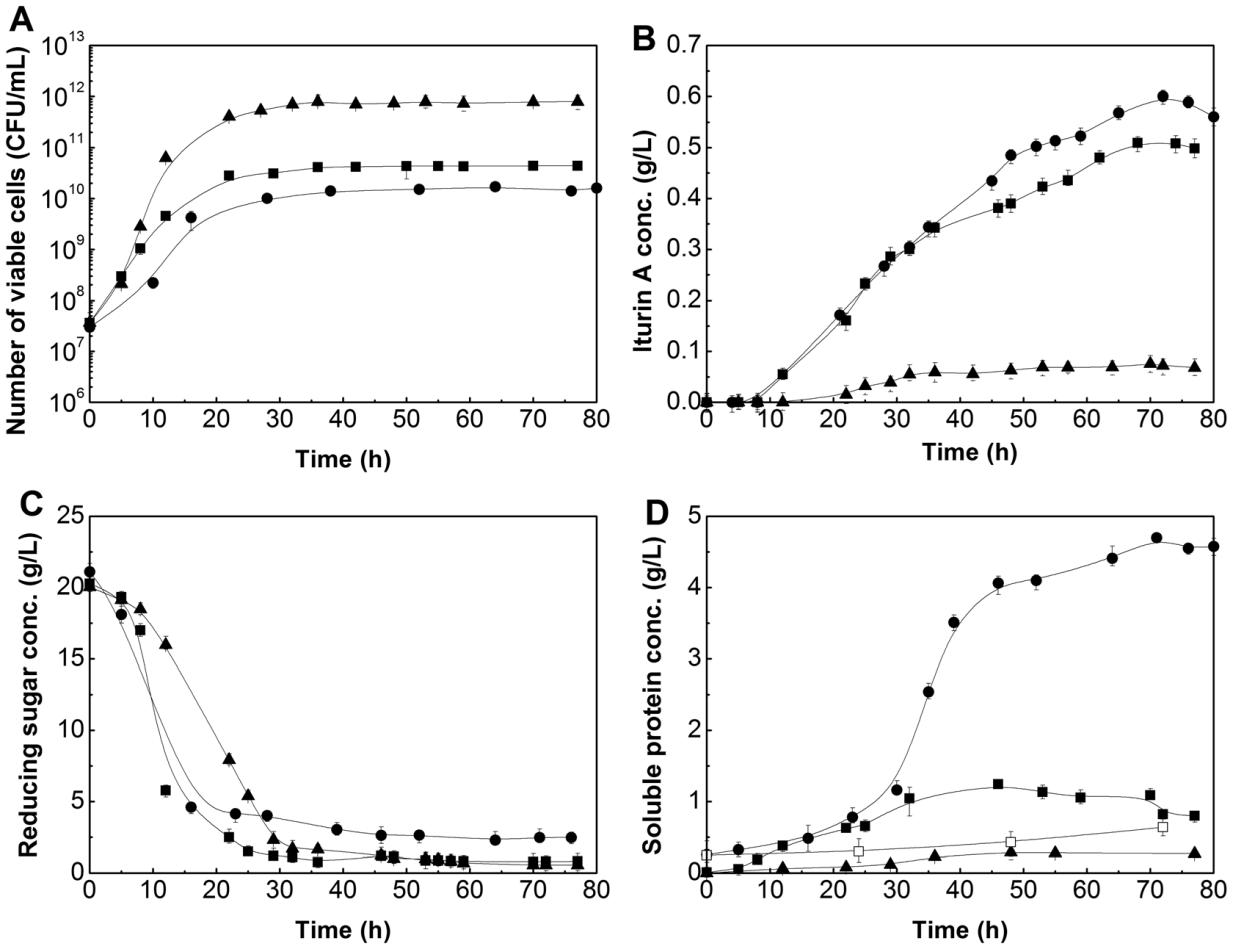
Time courses of the number of viable cells (A), iturin A concentration (B), reducing sugar (C) and soluble protein concentration (D) with different nitrogen sources in bioreactor batch fermentation. •: rapeseed meal; ▪: peptone; ▴: ammonium nitrate. Error bars represent the standard deviation of the means (N = 3).

To further investigate the application potential of low-cost rapeseed meal as nitrogen source on iturin A production, two different commercial nitrogen sources, peptone and ammonium nitrate, were applied in batch fermentations to compare the production performance of iturin A with that produced from rapeseed meal in a 7-L bioreactor. The optimal concentrations of peptone and ammonium nitrate were finalized at 30 and 8 g/L, respectively, based on our repeated flasks fermentation results (data not shown). As shown in Figure 3, when using ammonium nitrate as nitrogen source, the highest viable cells number reached 7.9×10^11^ CFU/mL, which was 18 and 46 fold higher than that when the peptone and rapeseed meal were used as nitrogen sources, respectively (Figure 3A); however, the iturin A production was the lowest and the maximum iturin A concentration was only 0.06 g/L (Fig. 3B). Among the three nitrogen sources, rapeseed meal was most suitable for iturin A production, the highest iturin A concentration reached 0.60 g/L at 72 h, which was 20% and 10-fold higher than that when peptone and ammonium nitrate were used as nitrogen source, respectively. This result indicates that nitrogen source from complex origin such as rapeseed meal and peptone well promoted the production of iturin A whereas the inorganic ammonium nitrate did not. Clearly, the N-availability of organic or inorganic origins has played different roles in iturin A synthesis. It is postulated that the inorganic N is available to increase the cell numbers but may not last for supporting the formation of secondary metabolites including iturin A in later phases. This finding is consistent with the result reported by Mizumoto and Shoda [30].

A significant decline of reducing sugar concentration was observed at the first 30 h for all fermentations, and then they remained almost constant during the later period (Figure 3C). When using peptone and ammonium nitrate as nitrogen sources, the stable reducing sugar concentrations after 30 h were close to 0 g/L, indicating that the reducing sugars were almost completely consumed. However, when rapeseed meal was used, the reducing sugar after 40 h remained at a relatively higher level of about 2.4 g/L till the end of fermentation, showing that there may be some unavailable sugar released from rapeseed meal. At the first 25 h, the soluble protein concentrations remained at 0.3–0.5 g/L and increased quickly from 0.5 to 4.0 g/L during 25–45 h when rapeseed meal was used as a nitrogen source. During 45–80 h, the soluble protein concentrations increased gradually and finally reached a constant level of about 4.5 g/L, which was 4.5 and 20 fold higher compared to that when peptone and ammonium nitrate were used as nitrogen sources, respectively (Figure 3D). To determine whether the increase in soluble protein was attributed to cultivation time, a parallel control experiment without inoculation was conducted. The soluble protein in the control sample increased slightly from 0.25 g/L at 0 h to 0.64 g/L at 72 h (Figure 3D), much lower than that of the treated, showing that the substantial increase of soluble protein was triggered by *Bacillus subtilis* fermentation.

Secondary metabolites, such as iturin A, are generally produced after a logarithmic growth phase in which nutrients become scarce [8]. In submerged fermentation, when using soluble organic and inorganic nitrogen sources, the nutrients of both carbon and nitrogen distributed abundantly in the liquid medium, and sufficient nutrients resulted in *Bacillus subtilis* growth rather than the production of iturin A (Figure 3A and B). However, compared to peptone and ammonium nitrate, the release and supply of nitrogen from rapeseed meal was slower and more favorable for iturin A production. Additionally, *Bacillus subtilis* regulated the nitrogen demand according to the available sugar in broth when using insoluble rapeseed meal as a nitrogen source. When *Bacillus subtilis* suffered from carbon starvation at about 20 h, the soluble protein increased rapidly (Figure 3C and D), indicating that the lack of available carbon source accelerated the release of soluble protein from rapeseed meal, and a portion of increased soluble protein may be used as a carbon source to offset the lack of sugar in the broth. This implied that the protease secreted by *Bacillus subtilis* strain played an important metabolic role.

### Obvious inducing effect of rapeseed meal on protease and cellulase secretion

**Figure 4:**
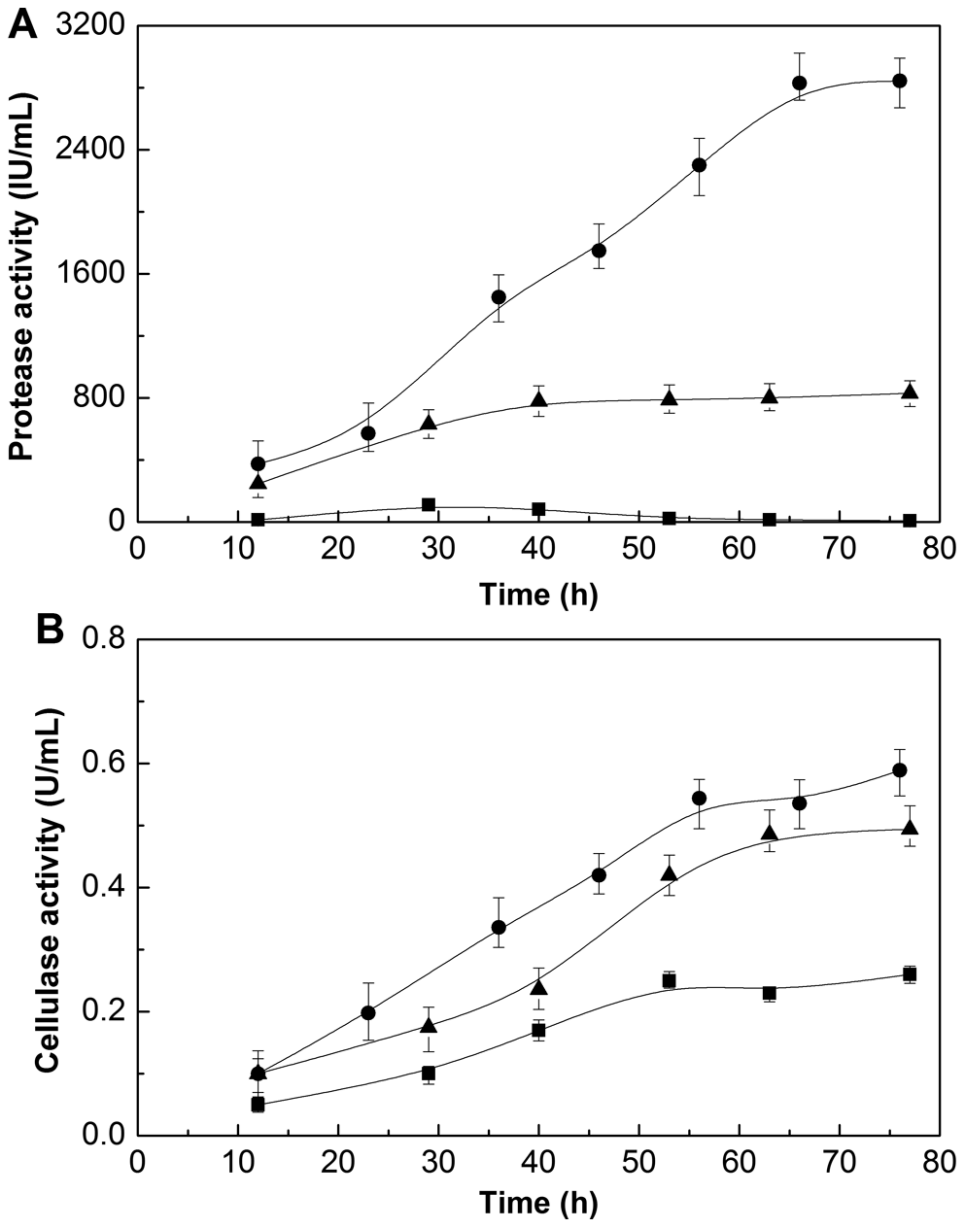
Time courses of protease and cellulase concentrations with different nitrogen sources in bioreactor batch fermentation. •: rapeseed meal; ▪: peptone; ▴: ammonium nitrate. Error bars represent the standard deviation of the means (N = 3).

The above results indicated more direct or efficient nitrogen assimilation from soluble peptone and ammonium nitrate resulted in faster growth of *Bacillus subtilis* compared to rapeseed meal (Figure 3A). Rapeseed meal provided an effective nitrogen source with low water solubility, so the release and utilization of rapeseed meal protein greatly depended on the degradation of proteases secreted by *Bacillus subtilis*. Moreover, both the cell growth and product iturin A synthesis relied on the continuous supply of available nitrogen from solid rapeseed meal. Therefore, protease activities from different nitrogen sources were compared during the fermentation. Figure 4A indicates that the secretion of protease was influenced greatly by the origin of nitrogen source, and there was an obvious induction effect of rapeseed meal on protease secretion. When rapeseed meal was used as a nitrogen source, protease increased with culture time, and the highest protease activity reached 2500 IU/mL at 70 h, which was 5 fold higher than that grown with peptone as the nitrogen source (Figure 4A). Interestingly, there was nearly no protease secretion when using ammonium nitrate as a nitrogen source.

On the other hand, the release of soluble protein may also be influenced by the high content of fiber in rapeseed meal, as it will pack and prevent the release and dissolution of protein from the rapeseed meal. Therefore, we analyzed and compared the cellulase activities under different nitrogen sources. When using rapeseed meal as the nitrogen source, the cellulase activities were the highest throughout the fermentation (Figure 4B), suggesting that rapeseed meal also has certain inducing effects on the secretion of cellulase and a portion of reducing sugar may be obtained from rapeseed meal when *Bacillus subtilis* suffered from carbon scarcity. A higher remaining reducing sugar concentration is likely from the hydrolysis of polysaccharide such as the cellulose in rapeseed meal by cellulase (Figure 3C).

### General change modes of on-line pH and dissolved oxygen in batch fermentation with different nitrogen sources

**Figure 5:**
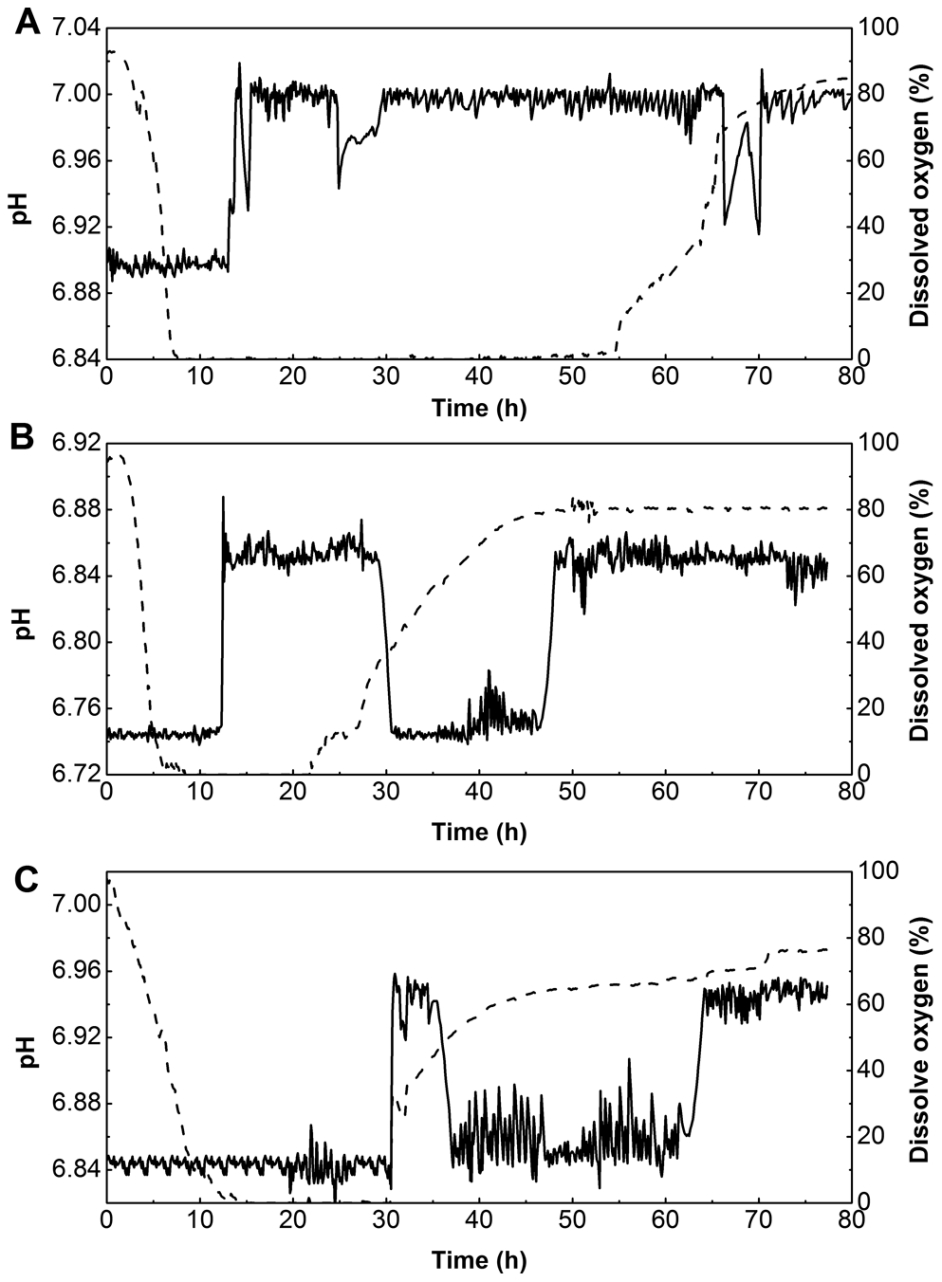
Time courses of pH and dissolved oxygen under different nitrogen sources during the entire fermentation process in bioreactor batch fermentation. A: rapeseed meal; B: peptone; C: ammonium nitrate; dot lines: Dissolved oxygen; bold lines: pH.

Currently, dissolved oxygen (DO) and pH are two conventional state variables for monitoring fermentative process, and to some extent, their changes reflect the metabolic characteristic of cells and may provide some useful information for industrial process control. The change modes of on-line pH and DO in batch fermentation under different nitrogen sources are shown in Figure 5. For all three batches, the aeration rate (2 vvm) and agitation speed (600 rpm) were held constant during the entire fermentation process. The actual pH was maintained automatically at approximately 7.0 by the addition of 4 mol/L NaOH and 4 mol/L H_2_SO_4_. Figure 5 indicates that DO and pH displayed different change tendencies when different nitrogen sources were used for iturin A production. When rapeseed meal was used, DO decreased to about 0% rapidly for the first 10 h and maintained almost constant until 55 h, indicating a high oxygen demand during this period. Further, DO began to rise gradually at 55 h with the depletion of available nutrients. On the other hand, pH was at the low limit for the first 12 h and then rose rapidly and was maintained at the high limit by continuous addition of acid solution till the cessation of fermentation (Figure 5A), indicating that alkali was added during the first 12 h and both acidic and alkali solutions were needed to maintain pH constant during the fermentation. When peptone was used as a nitrogen source, DO began to rise at 20 h, much earlier than that when using rapeseed as a nitrogen source (Figure 5B); moreover, the pH showed an intermittent change during fermentation: from the low limit (0–12 h), to the high limit (12–30 h), then to the low limit (30–50 h) again, and finally to the high limit (50–77 h). However, when inorganic ammonium nitrate was used as a nitrogen source, the sudden rise of pH and DO at 30 h was simultaneous, indicating that the available sugar was completely consumed at that time (Figure 5C and Figure 3C). In contrast to ammonium nitrate, using peptone and rapeseed meal protein as nitrogen sources might be used as a carbon source for iturin A synthesis when *Bacillus subtilis* suffered from carbon starvation. The differences in pH during the fermentation process may reflect different regulatory mechanisms of *Bacillus subtilis* in response to different nitrogen sources. The intermittent pattern of pH was only observed with strain grown on peptone and ammonium nitrate but not in rapeseed meal. The precise mechanism for this phenomenon remains elusive.

### Evaluation of protein and major anti-nutritional components of rapeseed meal before and after submerged fermentation

When rapeseed meal was used as a fermentative nutrient material for submerged fermentation, the related studies were mainly focused on its availability and the yield of target products. Importantly, rapeseed meal contains some toxic components, particularly glucosinolate and its degradation products oxazolidine (OZT) and isothiocyanate (ITC), which could interfere with the function of thyrold gland and adversely affect growth performance [31]. Therefore, for special bio-products with medical purposes or food applications, the distribution and degradation of toxic components in supernatant may be a factor to be considered when using rapeseed meal as fermentative material. The fermented sample analysis indicated that there were still a large portion of dry solid residues (about 4%, w/v) left at the end of fermentation (80 h). The fermented rapeseed meal residue containing *Bacillus subtilis* may be an ideal protein resource for animal feed via suitable dry processing, so the main anti-nutritional factors in residue should also be evaluated in addition to protein content. Thus, the changes of protein and major anti-nutritional components including crude fiber, sinapine, glucosinolate and its degradation products, isothiocyanate and oxazolidine thione, were determined and compared before and after fermentation. The initial sample data from Table 1 showed that most of the measured anti-nutritional components from raw rapeseed meal distributed both in the supernatant and the residue, and obvious losses for sinapine (40%) and glucosinolate (58%) were observed following sterilization. Additionally, the distribution of sinapine and glucosinolate in the supernatant versus residue were markedly different. The ratios of initial sinapine and glucosinolate in supernatant to residue were 1.45 and 6.67, respectively. After 72 h of fermentation, all of the measured anti-nutritional components both in the supernatant and the residue decreased significantly except for crude fiber (Table 1). The increased fiber content observed in fermented residue may be ascribed to the substantial release and utilization of protein from solid rapeseed meal. The sinapine contents in supernatant and residue decreased 97.8% and 93.3%, respectively, and the glucosinolate in supernatant and residue reduced 80.2% and 35.1%, respectively. The protein content in the residue reduced from the initial 41.6% to 37.8% at 72 h (Table 1), indicating that there were still a large number of proteins retained in the fermented rapeseed meal residue. Moreover, compared to raw rapeseed meal, both toxic components of isothiocyanate and oxazolidine thione in residue decreased greatly, and the remaining isothiocyanate was almost undetectable, suggesting that the remaining residue after fermentation may be used as a favorable, eco-friendly supplementary protein source for animal feed.

**Table 1:** Comparison of protein and major anti-nutritional factors both in supernatant and residue before and after fermentation.

Components	Raw material	Initial sample (0 h)		Fermented Sample (72 h)	
		Supernatant	Residue	Supernatant	Residue
Protein (%^a^ or g/L^b^)	42.4±0.1	0.40±0.02	41.6±0.2	4.5±0.1	37.8±0.3
Crude fiber (%)	18.2±0.2	-	17.4±0.3	-	27.8±0.1
Sinapine (µg/g^a^ or µg/mL^b^)	5882.6±3.2	187.9±0.3	1443.4±1.2	4.1±0.1	96.6±1.5
Glucosinolate (µmol/g^a^ or µmol/mL^b^)	13.94±0.1	0.46±0.02	0.74±0.01	0.093±0.03	0.48±0.02
Isothiocyanate (mg/g^a^ or mg/mL^b^)	1.66±0.2	0.028±0.001	ND	0.011±0.001	ND
Oxazolidine thione (mg/g^a^ or mg/mL^b^)	0.84±0.05	0.033±0.002	0.14±0.01	0.015±0.001	0.12±0.01

a The concentration units of components in supernatant.

b The concentration units of components in raw material or residue. ND: Not detected.

“-”: Not measured.

## Conclusions

Overall, this study explored the feasibility and effectiveness of untreated rapeseed meal as a nitrogen source for iturin A production by *Bacillus subtilis* 3–10 in submerged fermentation. Rapeseed meal was shown to produce a high amount of iturin A up to 0.60 g/L, much higher than that produced from peptone and ammonium nitrate media. Meanwhile, a significant inducing effect of rapeseed meal on protease secretion was found to ensure continuous supply of available nitrogen for iturin A synthesis. This approach may have potential in cost-effective production of iturin A and other valuable secondary metabolites using by-products of the rapeseed industry.
